# A Selective and Fast Approach for Volatile Metalorganics Assaying in Wastewater

**DOI:** 10.3390/molecules30051111

**Published:** 2025-02-28

**Authors:** Krzysztof Jankowski, Monika Truskolaska, Magdalena Borowska, Jacek Giersz, Edward Reszke

**Affiliations:** 1 Faculty of Chemistry, Warsaw University of Technology, ul. Noakowskiego 3, 00-664 Warsaw, Poland; truskolaska.monika@gmail.com (M.T.); magdalena.borowska@pw.edu.pl (M.B.); jjgiersz@gmail.com (J.G.); 2 Ertec-Poland, ul. Kłopockiej 13, 54-530 Wrocław, Poland; ertec@wp.pl

**Keywords:** volatile metalorganics, wastewater, screening, HSSPME, MIP-OES

## Abstract

A fast and green approach for the non-chromatographic assaying of volatile metalorganic compounds (VMOCs) is presented, involving the use of thermal desorption microwave-induced plasma optical emission spectrometry for the multi-species simultaneous determination of VMOCs in wastewater plant samples after headspace solid-phase microextraction (HSSPME-TD-MIP-OES), and optimized as a tool for the assessment of ambient exposure to hazardous VMOC pollutants. With the aim of VMOC monitoring, all species are separated and quantified within 10 s in comparison with about 10–20 min required by conventional GC-based procedures. Calibration against aqueous standards was carried out for several metalorganic species. The method was successfully applied for the quantitative extraction of As, Bi, Hg, Sb, Si and Sn compounds. Limits of detection ranging from 5 to 30 ng L^−1^ and relative standard deviations lower than 4% were obtained. The method is appropriate for high-sample-throughput measurements, and it proved to be suitable for the analysis of wastewater and sewage sludge samples.

## 1. Introduction

The quality of wastewater is generally assessed using a series of physical, chemical and microbiological tests. While metal content is widely established in the liquid phase [1,2], by contrast, the release of volatile compounds is scarcely studied. Apart from a multitude of volatile organic compounds, wastewater samples usually contain volatile metalorganics (VMOCs) and element–organic compounds like methylsiloxanes (VSiOCs). They are produced in wastewater environments either due to chemical transalkylation or due to biologically mediated methylation and hydride formation [3,4]. There are multiple pathways for methylation under wastewater treatment conditions. Several volatile organic species, e.g., methyl iodide (CH_3_I), acetic acid and ethyl acetate, exhibited good methylation of metal ions. A number of VMOCs were detected in the air surrounding the wastewater treatment plant [5,6,7]. The main problem of the multi-element speciation of non-volatile methylated metal(loid) species is the heterogeneity of the physicochemical properties of these compounds.

The presence of VMOCs in the environment has increased in the last few decades due to anthropogenic activities. These compounds appear as top hazard substances in the priority pollutant lists of the European Union [8]. Owing to the high toxicity of these compounds, even at the low concentrations found in WWTP samples, they should be monitored due to the long-time exposure of WWTP staff. Consequently, the screening of these compounds in the wastewater and sewage sludge is essential in order to assess the risks of a hazardous pollution event. Wastewater treatment processes have the function of reducing harmful pollutants in the wastewater. Unfortunately, these pollutants usually accumulate in sewage sludge, which acts as a long-term repository and helps to maintain their persistence in the WWTP environment. Thus, the health risk to workers from ambient volatile organic compound exposure must be assessed due to the low-temperature volatilization of VMOCs under WWTP conditions [4,9].

Gases released from the wastewater can be detected in the headspace of most of the liquid and solid media throughout the WWTP. In this case, methyl and hydride derivatives of mercury, arsenic, antimony and tin represent the major portion of the VMOCs [5,6]. Moreover, during anaerobic waste degradation, volatile silicon organic compounds (VSiOCs), including methylsiloxanes, are generated [7,10]. Owing to the high toxicity of organometallic compounds, fast, low-cost and multi-species detection techniques are necessary for screening analysis. For the multi-component or chemical speciation analysis of VMOCs, chromatographic methods are commonly used combined with the preconcentration procedure of cryogenic trapping all the compounds simultaneously [11,12]. In addition, the state-of-the-art analytical methodology currently available to release the ionic species of different metal(loid)s from the liquid or solid phase is derivatization by hydride generation (HG) [13,14].

One of the foremost methods is based on gas chromatography coupled with inductively coupled plasma mass spectrometry (GC-ICP-MS), which allows the speciation of VMOCs [11,13,15]. Alternatively, GC-MS/OES can be used as a detection coupling, in which the VMOCs’ identification is allowed by the mass spectrometer, whereas their quantification is performed by optical emission spectrometry [14]. Zeng [16] used a compact GC system to separate VMOCs, which was coupled with an atomic fluorescence detector to perform quantification. In commonly used coupled systems, the presence of a chromatographic column and the column–plasma interface seriously affects the transport efficiency and even causes inaccurate results due to the adsorption, condensation, artifact formation and transmethylation of analyte species. Therefore, to avoid this and to build a compact system, we designed the system without the interface between the TD unit and the subsequent MIP cavity with helium plasma.

A sample preparation step providing the isolation and enrichment of analytes is usually required to ensure sufficient sensitivity of a procedure. Modern approaches to VMOC sample preparation are dedicated to solventless methods. Solid-phase microextraction (SPME) was successively used for the determination of VMOCs, thus achieving efficient separation of the analytes from complex matrices and improving sample introduction efficiency [15,17]. The volatile species may simply be directly collected on the SPME fiber or may be liberated from liquid and solid samples by derivatization, which is followed by introduction into thermal desorption devices coupled to gas chromatographs. Some success in speciation by vapor-phase sample introduction into an MIP-OES was reported [18,19]. However, it was established that even the gentle thermal desorption of analytes was not sufficient for the baseline separation of different species, and, thus, it was combined with short-column chromatography [17,18].

Alternatively, accurate and precise analytical results by HSSPME-TD-DP-OES can be obtained based on the measurements of sharp transient signals produced by fast thermal desorption and the transport of volatile species through a miniaturized analyzer system. In the development of this green procedure, an effort was made to diminish sample manipulation and the use of other types of equipment in order to decrease any potential sample contamination and analyte loss. The main benefits of the proposed methodology compared to others are lower sample volumes and the faster and more environment-friendly extraction approach. The single-wavelength transient signals are easily extracted from the data set.

The objective of this study was to develop a method for assaying VMOC species by HSSPME separation with or without derivatization and subsequent determination by TD-MIP-OES. It was hypothesized that OES operated in simultaneous but high-frequency time-resolved analysis mode would be used to detect individual VMOCs separately directly after thermal desorption of the analytes from the SPME fiber. The potential for the co-elution of different VMOC species necessitates the use of a multi-element detection system. To validate the concept, we first determined both the amount of mercury and the concentration of methylmercury released. We then applied these findings to the development of speciation analysis given that the total metal(loid) content is typically divided into two or three defined species.

For multi-element speciation, the hydride generation method was employed as a preliminary derivatization step, given its efficacy in differentiating volatile hydrides of various elements based on their respective boiling point values. This approach was particularly advantageous for the analysis of non-volatile methylated species and inorganic species of As, Se, Sn, Sb, Te, and Hg.

## 2. Results

### 2.1. Optimization of the Separation and Detection Conditions

In order to optimize the SPME method, metal-containing solutions were analyzed using different exposure times, exposure temperatures and concentrations of matrix components including derivatizing agent (NaBH_4_) and mineral acid. For the HS sampling of inorganic compounds of Hg and As, reasonably short fiber exposure times of up to 5 min are adequate [20]. In turn, for VMOCs, a much longer sorption within 15–30 min is required [15,18]. For multi-species preconcentration, the 20 min long fiber exposure time was selected as the optimal duration for the extraction process. Carboxen has been identified as the most effective coating for the sorption of VOMCs.

To access the efficacy of SPME sampling for selected VMOCs, the experimental extraction efficiency (EE) has been determined according to Haberhauer Troyer et al. [21]. For a given metalorganic species, the EE percent value has been calculated based on the results of n successive extractions of the same sample after the derivatization step, according to the following equation:

(1) $${\text{logPA}}_{n}=\left(n-1\right)\cdot \text{log}\left(1-\frac{\text{EE}}{100}\right)+{\text{logPA}}_{1}$$

where PA_n_—peak area for a given sorption repetition, EE—percent extraction efficiency, and PA_1_—peak area for the first sorption process.

The experimental extraction efficiency percent values of 85, 73, 53.5, 60 and 52 have been determined for mercury vapor, stibine, methylmercury chloride, tetraethyllead and dibutyltin dihydride, respectively.

In the developed TD-MIP-OES system, sample transport is carried out through a heated quartz transfer line, which offers several advantages. This solution ensures continuity of the connection and provides inherent heating of the transfer line. Additionally, the relatively short distance between the plasma and the sampling port minimizes adsorption of the analytes on the quartz tube walls, which is particularly crucial in the case of chemically active compounds such as the VOMCs. The application of helium gas, the gas that exhibits better thermal conductivity in comparison to argon, ensures optimal kinetics for sample evaporation. A notable benefit of the MIP lies in its ability to generate low-volume plasma, which is a feature that prevents the distortion of sharp analytes zones, ensuring the integrity and precision of the analytical data. The high elemental selectivity and the rapid response of the OES detector serve to minimize background even in the presence of complex samples. Consequently, the limits of detection for metals are relatively low, ranging from 3 to 200 ng L^−1^.

The goal of optimization was to achieve a separation of all VMOCs within approximately 10 s. The parameters to be optimized include the sample desorption temperature, the carrier gas flow rate and the signal integration time. The limitations to be considered during the optimization process included the following: (1) low plasma stability at helium flow rates below 100 mL min^−1^; (2) substantial differences between boiling point values for analytes; and the (3) acquisition of a representative number of data points for a short transient signal.

**Figure 1:**
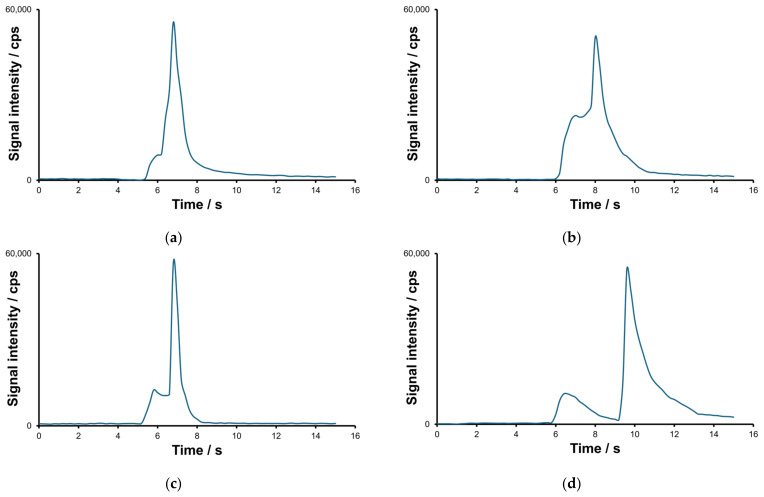
Effect of desorption temperature (T) and helium flow rate (F) on the separation of mercury compounds: (**a**) T = 175 °C and F = 0.35 mL min^−1^; (**b**) T = 200 °C and 0.35 mL min^−1^; (**c**) T = 250 °C and F = 0.25 mL min^−1^; (**d**) T = 250 °C and F = 0.35 mL min^−1^.

The separation of the VMOCs depends on the carrier gas flow rate and desorption temperature. The inter-element separation is facilitated by the high elemental selectivity of the detector. It is imperative to consider the interdependence of certain variables involved in TD-MIP-OES. The operational temperature range of the TD unit is thus determined to be between 100 and 250 °C. The He flow rates for analytes transport range from 150 to 300 mL min^−1^, matching the requirements for stable operation of the MIP. The carrier gas flow rate primarily affects the retention time of analytes and the analytes separation efficiency. For all analytes, the He flow rate at 250 mL min^−1^ and the desorption temperature of 250 °C were selected. The influence of the essential experimental parameters on the separation is exemplified in Figure 1.

Under the optimized conditions, the typical half-width of the peak is between 0.4 and 0.8 s. To ensure the acquisition of a sufficient number of data points for short transient signals, the signal integration time has been optimized, and a duration of 200 ms has been determined to be the most suitable. However, due to the proximity of the boiling points, it has been unfeasible to ascertain baseline separation conditions.

### 2.2. Analytical Performance

A series of validation studies were conducted, employing standard solutions of various analytes to determine the most important analytical parameters. These parameters encompassed precision, linearity and limits of detection.

Under the optimized experimental conditions, the limits of detection were determined to be 6.7 for MeHgCl, 5.2 for Hg, 16 for Bu_3_SnCl and 25 ng L^−1^ for SbH_3_, respectively (see Table 1). The calibration curves for Hg, MeHgCl, SbH_3_ and Sn derived from the signal intensities of the prominent spectral lines of the respective metal(loid)s cover three orders of magnitude in analyte concentration. The linearity of the curves, as indicated by correlation coefficients greater than 0.99, was found to be satisfactory for all analytes at trace concentration levels.

The analytical validation of the methodology for screening VMOCs is finally conducted through the use of standards and certified reference materials, including assays on water and sediment matrices, respectively. As demonstrated in Table 2, the recoveries (n = 3) were satisfactory (≥95%) for all analytes.

### 2.3. Analysis of Samples from WWTP

**Figure 2:**
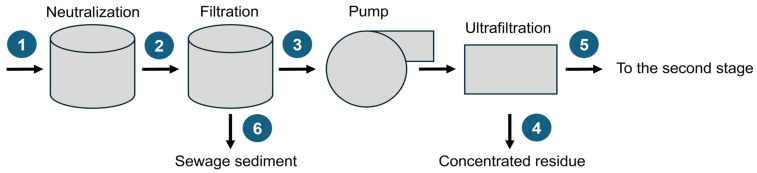
The scheme of primary stage of the wastewater treatment process plant.

The method described herein was employed for the identification and semi-quantification of VMOCs released from chemical wastewater and sewage sediment in the primary stage of the wastewater treatment process. The treatment train consisted of primary clarifiers that included filtration and ultrafiltration (Figure 2). Four wastewater samples and two sewage sediment samples were collected from a WWTP and analyzed in duplicate.

Following microwave digestion, the samples were analyzed using inductively coupled plasma optical emission spectrometry. This analytical approach provided the total element concentration for both each element and each sample of interest, as shown in Table 3.

Both the analytes preconcentration process and low-flow separation in combination with multi-element detection enables the direct analysis of several VMOCs, obviating the need for derivatization. For the analysis of wastewater samples or sediments, sample manipulations were generally performed inside a SPME vial, thereby establishing equilibrium conditions [22]. The measurements were taken under gas–liquid (solid) equilibrium conditions to characterize the release of VMOCs at the designated point in time. In the initial phase, the release of dissolved volatile compounds occurred without derivatization (e.g., Me_2_Hg, MeI, L2). Subsequently, a hydride generation methodology was employed by treating 10 mL of sample wastewater with 1 mL of 1 M HCl, which was followed by the addition of 1 mL of a freshly prepared 5% solution of NaBH_4_.

High-resolution element signal intensity–time profiles were recorded for the elements of interest using a multi-channel OES detection system. As illustrated in Figure 2, the sewage gas contains many species, and the related peaks are not baseline separated in the TD-MIP-OES system.

**Figure 3:**
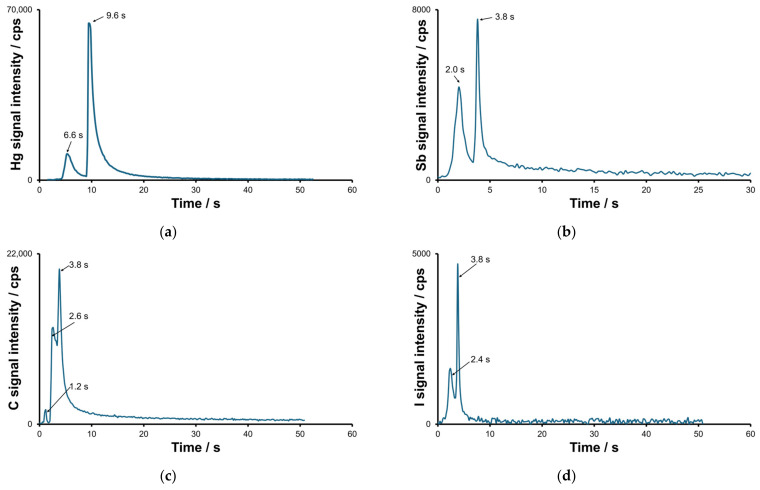
Hg (**a**), Sb (**b**), C (**c**) and I (**d**) signal intensity–time profiles recorded involving the multi-channel OES detection system.

For Hg, the higher broadened peak corresponds to semi-volatile elemental mercury (t_r_ = 9.6 s), and the lower (t_r_ = 6.6 s) to methylmercury chloride, as evidenced by a comparison of the retention times with those of standards (Figure 3a). Apart from mercury species, which were recorded in all gas samples, other species were observed in sewage gas. For antimony and iodine, at least two different volatile compounds with similar retention times were detected in selected gaseous samples. For tin, one major peak was detected in the influent sample, which was assumed to be Me_4_Sn.

The speciation analysis of a given element was achieved by single channel detection, while the simultaneous element-selective detection of species for a variety of elements was performed by multi-channel detection.

**Figure 4:**
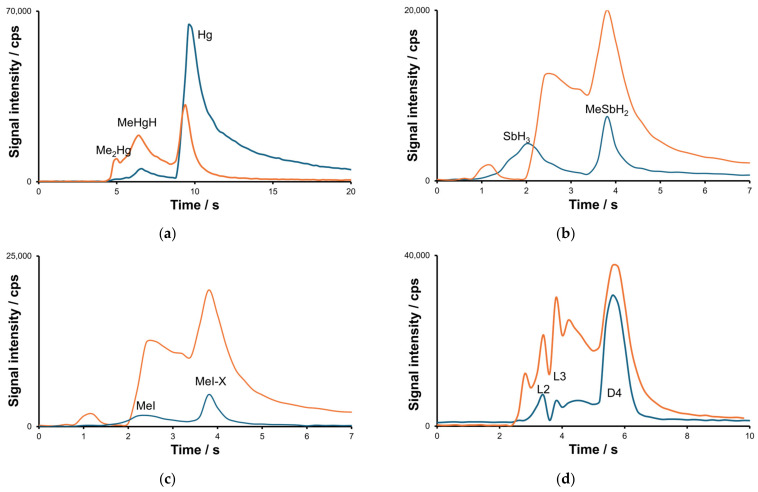
The transient signals recorded for Hg (**a**), Sb (**b**), I (**c**) and Si (**d**) species (blue line). Orange line shown at each graphs represents signals recoded for carbon.

At least three different volatile mercury compounds were detected in the gaseous samples (Figure 4a). For two of these peaks, designated Me_2_Hg and MeHgH, the retention times and peak shapes for Hg and C profiles were found to be well correlated. The most volatile species was identified as dimethyl mercury based on the results of the identification studies described below. For Sb and I, two species were detected for each element, as displayed in Figure 4b,c. The identification of stibine and methyl iodide was based on their previously determined retention time values, which were in turn determined for related standards. Identification tests for MeSbH_2_ and MeI-X are described below. The presence of methyl iodide in the wastewater media implies the occurrence of an intense methylation process.

The primary results of this study indicate the presence of methylsiloxanes in selected wastewater samples with octamethylcyclotetrasiloxane (D4) emerging as the predominant compound. The similarity in shapes observed for carbon and silicon profiles is clearly seen in Figure 4d. For a more comprehensive identification process, please keep following this section.

**Figure 5:**
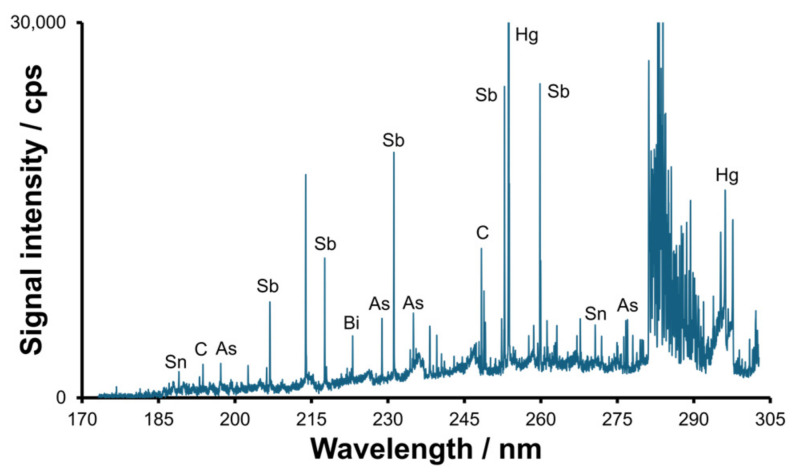
The spectrum for influent obtained using HG SPME-TD-MIP-OES technique.

The use of the hydride generation technique for the derivatization of metal(loid) compounds in the wastewater samples has enabled the detection of ionic metalorganic compounds that are incapable of forming volatile species. The headspace SPME method was utilized for the extraction and preconcentration of the target species from the sample following derivatization with sodium tetrahydroborate. The spectrum obtained for the influent sample after hydride generation and headspace multi-element separation/preconcentration of the formed hydrides on the SPME fiber is displayed in Figure 5. The presence of multiple spectral lines of As, Bi, C, Hg, Sb and Sn revealed the formation of inorganic or metalorganic hydrides of the elements.

**Figure 6:**
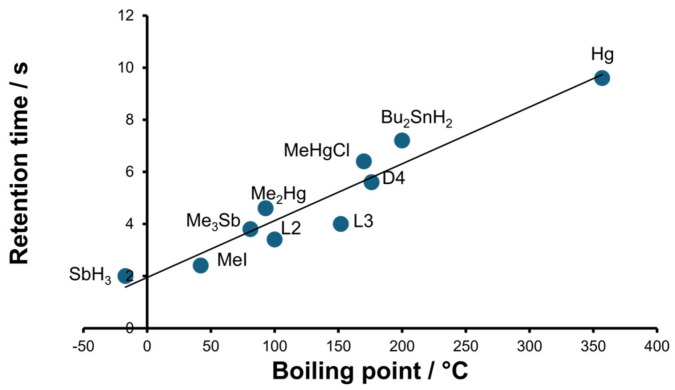
Correlation of the boiling points and retention times of the standards and identified species for the proposed HSSPME-TD-MIP-OES method.

In order to identify the species detected and to demonstrate the robustness of the proposed method, the correlation between the boiling points (bp) and retention times (T_r_) for the standards and identified species was examined (Figure 6). The linear relationship for a wide bp range between −17 °C and 357 °C was calculated to be

(2) $$T_{r}=0.0218\;\text{bp}+1.9489$$

with an acceptable correlation factor R^2^ = 0.9122. The linear correlation is in line with that obtained by Feldmann and Hirner [5] for the GC-ICP-MS method.

Due to the existence of a correlation, the presence of some retention times was indicative of unknown peaks. The peaks of Si (Figure 3d) could be identified as hexamethyldisiloxane (L2) and octamethyltrisiloxane (L3) with bp values of 100 °C and 152 °C, respectively.

**Figure 7:**
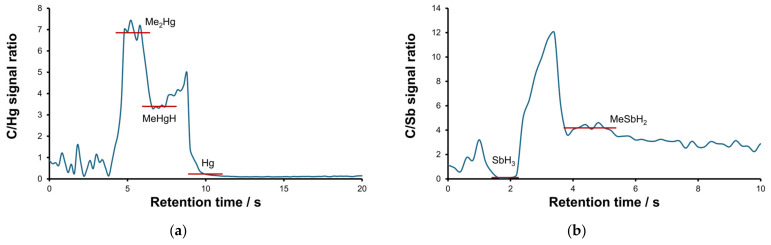

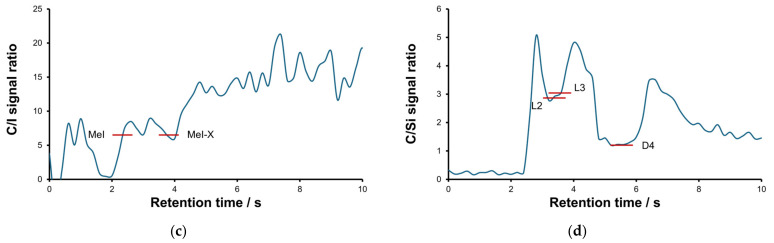
Carbon:element signal ratios calculated across entire signal intensity–time profiles for Hg (**a**), Sb (**b**), I (**c**) and Si (**d**).

The identification of detected species was continued by processing the post-measurement data to calculate carbon:element signal ratios and subsequently to approximate the stoichiometry of the species. The results for Hg, Sb, I and Si are shown in Figure 7. The ratio was calculated across the entire signal intensity–time profile, shown in Figure 4, after background subtraction. The time scans for the background were obtained separately for each element (the readout is typically five pixels left to the line peak position). It should be noted that the calculated ratio values are not explicitly the stoichiometry coefficients due to differences in the excitation efficiency of each element. The average value of the ratio was calculated in proximity to the retention time of each species, and it is marked in red.

The results obtained for Hg and Sb species are consistent and reliable. For inorganic species (Hg and SbH_3_), the ratio values are close to zero. For Me_2_Hg, the ratio value is twice as large as that for MeHgH. For iodine (Figure 7c), the ratio values for two compounds are close to each other, suggesting the formation of an unknown adduct MeI-X. As anticipated, the C:Si signal ratio values for two linear methylsiloxanes L2 and L3 are comparable. However, for cyclic species D4, the ratio value appears to be too low, necessitating further investigation to elucidate the observed discrepancy.

Finally, some efforts have been made to determine the concentrations of species identified in wastewater samples. The compound independent calibration technique, as discussed by Andersson [23], operated under the assumption that the sensitivities for all species of a given element are equal. In the context of MIP-OES, the feasibility of this technique has been validated for a number of homologous groups of low molecular weight organic compounds. Utilizing this technique, the other compounds of a given element have been semi-quantified, and the results are compiled in Table 4.

A total of five elements were detected in the gas phase in equilibrium with liquid and solid samples. The concentration of these elements was in the range between ng and µg per m^−3^ based on a semi-quantitative calibration method with MeHgCl, SbH_3_ and Bu_2_SnH_2_ as calibration standards. Elemental mercury was the predominant species, and it was detected in all samples analyzed at concentrations ranging from 10 to 6000 ng m^−3^. Dimethylmercury was also detected in all samples, except the effluent, in concentrations of 20–250 ng m^−3^.

Concentrations of Sb, Sn, As and I frequently reached levels of 850 ng m^−3^. The total Sb concentrations of the soil samples were generally higher than those of Sn or As, with the exception of influent, in which high amounts of inorganic As were determined. The methylantimony dihydride species was identified as the predominant species when expressed as a percentage proportion of the total Sb content. The VSbOs concentrations were found to be significantly higher compared to those of Sn and As, indicating that Sb undergoes facile methylation in wastewater media.

The concentrations of MeI in sewage sediment gas were calculated to range from 0.2 to 1 μg m^−3^. This indicated that the sediments possess a high potential for the site-specific release of VMOCs during the process of further landfilling.

The derivatization of samples via hydride generation revealed that elemental mercury was the predominant and easily generated species released from wastewater samples with at least 80% efficiency except in effluent. Among VMOCs, monomethyl Hg and Sb hydrides are predominant species with concentrations up to 2 µg L^−1^.

Methylsiloxanes were detected in all of the analyzed samples, with D4 being the predominant compound, exhibiting concentrations ranging from 0.40 µg L^−1^ to 11.6 µg L^−1^. L2 and L3 were found to be below the LOQ in the wastewater samples, with exception of the influent sample, yet they were detected in the sewage sludge samples. The mean concentrations of L2, L3 and D4 in sludge were determined to be 19 μg kg^−1^, 13 μg kg^−1^, and 26 μg kg^−1^, respectively. The results of this study indicate that more VSiOCs are transferred from the wastewater to the sludge due to their adsorption ability.

## 3. Materials and Methods

### 3.1. Solid-Phase Microextraction Device

The manual SPME extraction and preconcentration device was produced by Supelco, Bellefonte, PA, USA. The fiber coated with Carboxen, Stableflex 85 µm has been found to be the most effective for the sorption of a wide range of VMOCs. Twenty milliliters of glass vials closed with PTFE-coated silicon rubber septum were used for sampling. Proper mixing of the liquid sample solutions during the SPME process was achieved with a magnetic stirrer.

### 3.2. TD-MIP-OES Instrument

The TD-MIP-OES apparatus consists of a modified Beenakker-type cavity with a coaxial coupler and a power generator (Ertec-Poland, Wrocław, Poland) for sustaining the helium MIP, a laboratory-made thermal desorption unit, a gas flow module, and a compact OES spectrometer Ava-Spec 3648 USB2 (wavelength range 170–300 nm) with optical fiber FC-UV400-2SR-HT (Avantes, The Netherlands). A portable computer control system was used for direct and post-measurement data processing.

**Figure 8:**
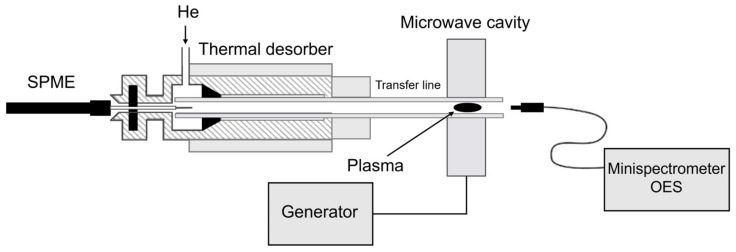
A schematic diagram of experimental setup.

The on-piece integrated line for analyte vaporization and transport consists of a temperature programmable electrically heated GC-type port sealed with silicone septum (Thermogreen, Supelco, Bellefonte, PA, USA) with a splitless injector coupled to the 2 mm i.d. 15 cm long quartz transfer line with the exit placed directly at the base of the plasma cavity to minimize sample dispersion. The coaxial coupling significantly has been shown to enhance both the plasma positioning and stability when compared with a typical Beenakker cavity, as previously described [19]. Helium was used as the carrier/plasma gas at a flow rate of 150–300 mL min^−1^. Figure 8 shows details of this setup.

The thermal desorption unit and plasma excitation source were made as a compact system capable of delivering volatile analytes released from the SPME fiber directly to the plasma emission detector within a few seconds. Subsequent to the thermal desorption process, volatile compounds were transferred to the plasma via a quartz transfer line, which was facilitated by the carrier gas. An optical fiber was used for the collection of the emission signal. As SPME is typically a solvent-free sampling technique, the entire volume of the desorbed analyte can be introduced into the plasma without causing any disturbance. Due to the small volume of the SPME extraction phase, sample desorption can frequently be accomplished expeditiously, and the analytes can be introduced in a very narrow zone. This, in turn, can lead to a substantial enhancement in measurement sensitivity. The operating conditions used for HSSPME-TD-MIP-OES are summarized in Table 5.

For samples digestion, a microwave unit Magnum II (Ertec-Poland, Wrocław, Poland) was used.

### 3.3. Reagents and Reference Materials

Inorganic mercury working solutions were prepared by appropriate dilution of the mercury standard solution for AAS, containing 1.000 mg mL^−1^ Hg as HgCl_2_ in 10% HNO_3_ from Sigma Aldrich, Warsaw, Poland. The working solutions of MeHgCl were prepared on a daily basis by diluting of the MeHgCl standard solution (1.000 mg L^−1^—calculated as Hg) in 0.5% acetic acid and 0.2% hydrochloric acid (Brooks Rand Lab, Seattle, WA, USA) in deionized water. These solutions were then stored in glass bottles at 4 °C.

Sodium tetrahydroborate was purchased from Sigma Aldrich, Warsaw, Poland, and a freshly prepared 5%*w*/*v* aqueous solution of NaBH_4_ was used as a derivatization agent for the determination of volatile hydrides. Deionized water was used throughout.

The reference materials, CRM-CC580 estuarine sediment and ERM-CA 011a hard drinking water were obtained from LGC Standards, Warsaw, Poland.

### 3.4. Procedures

Sampling procedure. Samples were collected from the first stage of treatment at the pilot wastewater treatment plant. Wastewater samples were collected without headspace in 1000 mL PET bottles and quickly sealed with PTFE-coated seals. Grab samples of wastewater sludge were taken from the sewage sludge tank outlet. Sludge samples were collected in glass containers. All samples were cooled to 4 °C.

In general, the HSSPME separation/preconcentration procedure is as follows: 10 mL volume of sample solution was placed in a septum-sealed 20 mL glass vial. The SPME fiber was subsequently inserted into the HS for a predetermined sampling time and temperature, the values of which were contingent upon the experiment. Thereafter, the fiber was withdrawn and transferred to the TD unit for TD-MIP-OES measurement.

Screening analysis of VOMCs directly released from sediment sample. The procedure for the screening and analysis of volatile organometallics in sediment samples entails the placement of 1.0 g of sediment in the 20 mL SPME via, which was followed by the solid-phase microextraction carried out at either room temperature or an elevated temperature. This process involves the exposure of the SPME fiber to the headspace for a duration of 30 min. The fiber is then withdrawn into the needle, and the SPME device is transferred into the TD port, which is heated to 175 °C for the introduction of the analytes into the analyzer.

Determination of non-volatile metal(loid)s ionic species in wastewater after hydride generation. In situ derivatization is a process that involves the addition of a 1 mL solution of 5% NaBH_4_ solution and 0.2 mL of concentrated hydrochloric acid to a 10 mL wastewater sample in an SPME vial. This process produces volatile hydrides, which are then used to determine the total volatile metal(loid) compounds present in the sample. The solution is stirred for 3 min using a magnetic stirrer. Next, the sorption procedure is carried out as described for direct VMOCs determination.

Microwave digestion of samples. An analysis of wastewater and sediment was conducted to ascertain the total concentrations of major and minor elements. This analysis was performed at various dilutions using inductively coupled plasma optical emission spectrometry. A representative 1–2 g wet weight sample was digested with the addition of trace metal grade nitric acid. Following the digestion, the samples were filtered and analyzed using a GBC Scientific Integra XL ICP-OES instrument. The resulting data are presented in Table 6.

## 4. Conclusions

The thermal desorption of VMOCs from SPME fiber has emerged as a promising microsample introduction technique for the TD-MIP-OES analysis of wastewater, offering a 100-fold increase in speed over commonly used gas chromatography-plasma spectrometric systems. The simultaneous detection of multiple Hg, Sb, Sn, Si As and I species within a span of 10 s is attainable through the utilization of straightforward instrumentation and minimal sample volume. This approach aligns with the principles of green chemistry. The HSSPME-TD-MIP-OES technique has been shown to exhibit high reproducibility with regard to retention times of individual VMOCs, is easy to work up and sensitive, offering limits of detection ranging from 5 to 30 ng L^−1^. The identification methodology can be useful when searching unexpected volatile VMOCs.

The compatibility of He flow rates with the plasma gas flow required for the detection of trace VMOCs in wastewater samples by MIP-OES makes it a suitable method for VMOCs speciation in wastewater samples at the level of tens of mg L^−1^ and even tens of µg L^−1^ for methylsiloxanes.

A considerable quantity of Hg and Sb VMOCs have been known to be released from wastewater media. Given their toxicity, occupational health and safety may not always be assured in the vicinity of wastewater treatment areas.

This work demonstrates that the combination of HSSPME and TD-MIP-OES allows for the rapid, simultaneous and reliable identification and semi-quantitative determination of a number of volatile metalorganics at trace levels in wastewater and sewage sludge samples.

## Figures and Tables

**Table 1:** Analytical figures of merit for determination of Hg, SbH_3_, MeHgCl and Bu_3_SnCl by HSSPME-TD-MIP-OES.

Parameter	Sn as Bu_3_SnCl	Determination of Sb as SbH_3_	Hg as MeHgCl	Hg as Hg^0^
Linear dynamic range [ng L^−1^]	50–20,000	75–10,000	20–20,000	20–50,000
Limit of detection [ng L^−1^]	16	25	6.7	5.2
Relative standard deviation %	3.5	3.8	3.1	3.3

**Table 2:** Analysis of Hg, MeHg, Sb and Se in certified reference materials using proposed method.

Sample	Hg_tot_ Found Value [mg kg^−1^]	Hg_tot_ Certified Value [mg kg^−1^]	MeHg Found Value [mg kg^−1^]	MeHg Certified Value [mg kg^−1^]	Recovery [%]
Estuarine sediment ERM CC580	128.7 ± 2.1	132 ± 3	0.081 ± 0.008	0.075 ± 0.004	97.5; 108
Wastewater sediment	22.5 ± 0.2	23.2 ± 0.3	0.161 ± 0.011	n.a.	97; -
	As_tot_ found value [ng mL^−1^]	As_tot_ certified value [mg kg^−1^]	Se_tot_ found value [mg kg^−1^]	Se_tot_ certified value [mg kg^−1^]	
Hard drinking water ERM-CA 011a	10.5 ± 0.9	10.1 ± 0.6	10.4 ± 0.7	10.7 ± 0.7	104; 97

Hg_tot,_ As_tot,_ Se_tot_—total concentration of the respective element.

**Table 3:** Total elements concentration determined in wastewater and sludge samples.

	1 Influent	2 Wastewater After Neutralization	4 Concentrate Residue	5 Effluent	6 Sewage Sediment
Analyte	Concentration/ppm				Content/mg kg^−1^
Bi	15	n.d.	n.d.	n.d.	n.d.
Pb	13.9	6.8	11.1	0.1	0.5
Sb	28.8	21.2	32.8	0.6	24
Sn	4.4	0.5	1.8	0.15	3
Hg	24.6	17.2	4.5	1.1	36
Te	0.25	0.1	n.d.	0.15	n.d.
Zn	220	50	65.5	1.3	63
Cd	30	29.7	25.8	1.1	117
I	25	22	1.5	0.1	62
As	9.4	2.1	3.1	0.2	-
Se	1.7	0.7	0.55	0.2	-
Fe	79	0.3	0.3	n.d.	70
Mn	106	38	36	0.4	-
Cu	36	35	34	27	78
Cr	45	28	40	0.2	42.5
Ni	57	25	32	0.3	34

**Table 4:** Identification and quantification of Hg, Sb, As and I species in wastewater samples.

Sample	Total Metal(loid) Concentration/mg L^−1^					Total VMOCs Concentration/ ng m^−3^					Identified Species
	Total Volatile Hydrides Concentration/mg L^−1^										
	Hg	Sb	Sn	As	I	Hg	Sb	Sn	As	I	
Influent	24.6	28.8	4.4	29.4	25.0	7200	n.d.	n.d.	n.d.	n.d.	Me_2_Hg, MeHgCl, Hg
	23.8	5.6	2.1	7.4	n.d.						Me_2_Hg, Hg, MeHgH, SbH_3_, MeSbH_2_, AsH_3_, SnH_4_
After neutralization	17.2	21.2	0.5	2.1	22.1	850	n.d.	n.d.	n.d.	n.d.	MeHgCl, Hg
	15.1	0.10	0.06	n.d.	n.d.						Me_2_Hg, Hg, MeHgH, SbH_3_, MeSbH_2_, SnH_4_
Concentrated residue	4.5	32.8	1.8	3.1	1.5	4700	60	n.d.	n.d.	n.d.	Me_2_Hg, MeSbH_2_, MeHgCl, Hg
	3.8	7.5	0.2	0.6	n.d.						Me_2_Hg, Hg, MeHgH, SbH_3_, MeSbH_2_, AsH_3_, SnH_4_
Sewage sludge 1	29 *	11 *	2.8 *	0.3 *	54 *	750	n.d.	n.d.	n.d.	250	Me_2_Hg, Hg, MeHgCl, MeI
	1.4 *	0.5 *	n.d.	n.d.	n.d.						MeHgCl, Hg, MeSbH_2_
Sewage sludge 2	36 *	24 *	3 *	n.d.	62 *	480	50	100	n.d.	850	Me_2_Hg, Hg, MeHgCl, MeI
	0.82 *	0.91 *	0.36 *	n.d.	1.4 *						Me_2_Hg, Hg, MeHgH, MeSbH_2_, AsH_3_,
Effluent	1.1	0.60	0.15	0.20	0.11	10	n.d.	n.d.	n.d.	n.d.	Hg
	0.32	n.d.	n.d.	n.d.	n.d.						Hg

* for sewage sludge, the element content is expressed in mg kg^−1^.

**Table 5:** Experimental conditions for HSSPME-TD-MIP-OES measurements.

Parameter	Value
Frequency	2.45 GHz
Applied power	50–150 W
Plasma configuration	Beenakker cavity with coaxial coupling
Fiber coating	Carboxen
Preconcentration time	20 min
Desorption temperature	150–250 °C
Carrier gas flow rate	150–300 mL min^−1^

**Table 6:** Operating conditions for ICP-OES measurements.

Parameter	Value
Applied power	1200 W
Height above coil	4 mm
Nebulizer gas flow rate	0.5 L min^−1^
Plasma gas flow rate	0.5 L min^−1^
Cooling gas flow rate	12 L min^−1^
Wavelength	Hg 253.6 nm Sb 259.8 nm Sn 180 nm As 234.9 nm I 206.1 nm Si 251.6 nm
Wavelength	Bi 223.1 nm Cd 226.5 nm Cr 205.6 nm Cu 324.7 nm Fe 238.2 nm Mn 259.4 nm Ni 232.0 nm Pb 220.3 nm Te 214.3 nm

## Data Availability

All data generated or analyzed during this study are available from the corresponding author on reasonable request.
